# Impact of Glucoraphanin-Mediated Activation of Nrf2 on Non-Alcoholic Fatty Liver Disease with a Focus on Mitochondrial Dysfunction

**DOI:** 10.3390/ijms20235920

**Published:** 2019-11-25

**Authors:** Liang Xu, Naoto Nagata, Tsuguhito Ota

**Affiliations:** 1 Key Laboratory of Laboratory Medicine, School of Laboratory Medicine and Life Science, Wenzhou Medical University, Wenzhou 325035, China; wanzhe1023@126.com; 2 Department of Cell Metabolism and Nutrition, Advanced Preventive Medical Sciences Research Center, Kanazawa University, Kanazawa, Ishikawa 920-8640, Japan; nnagata@staff.kanazawa-u.ac.jp; 3 Division of Metabolism and Biosystemic Science, Department of Medicine, Asahikawa Medical University, Asahikawa, Hokkaido 078-8510, Japan

**Keywords:** nonalcoholic fatty liver disease (NAFLD), insulin resistance, chronic inflammation, mitochondrial dysfunction, Nrf2, sulforaphane, glucoraphanin

## Abstract

Non-alcoholic fatty liver disease (NAFLD) is a common disease in Western nations and ranges in severity from steatosis to steatohepatitis (NASH). NAFLD is a genetic-environmental-metabolic stress-related disease of unclear pathogenesis. NAFLD is triggered by caloric overconsumption and physical inactivity, which lead to insulin resistance and oxidative stress. A growing body of evidence indicates that mitochondrial dysfunction plays a critical role in the pathogenesis of NAFLD. Mitochondrial dysfunction not only promotes fat accumulation, but also leads to generation of reactive oxygen species (ROS) and lipid peroxidation, resulting in oxidative stress in hepatocytes. Nuclear factor erythroid 2-related factor 2 (Nrf2) is an important modulator of antioxidant signaling that serves as a primary cellular defense against the cytotoxic effects of oxidative stress. The pharmacological induction of Nrf2 ameliorates obesity-associated insulin resistance and NAFLD in a mouse model. Sulforaphane and its precursor glucoraphanin are derived from broccoli sprouts and are the most potent natural Nrf2 inducers—they may protect mitochondrial function, thus suppressing the development of NASH. In this review, we briefly describe the role of mitochondrial dysfunction in the pathogenesis of NASH and the effects of glucoraphanin on its development.

## 1. Introduction

Non-alcoholic fatty liver disease (NAFLD) ranges from simple hepatic steatosis and nonalcoholic steatohepatitis (NASH) to cirrhosis [1,2]. Histologically, the key components of NASH are steatosis (e.g., hepatic triglyceride content greater than 5% of the hepatic volume or of the liver weight), hepatocellular ballooning, lobular inflammation, and fibrosis [3,4]. NAFLD is one of the most common chronic conditions in the United States and other developed countries [5,6]. The overall prevalence of NAFLD in Western countries is 20–50%, and reaches 75–90% among obese and morbidly obese individuals [6,7,8]. About 2–3% of patients with NAFLD develop NASH, and 5–8% of patients with NASH develop hepatic cirrhosis within five years [9]. Moreover, NAFLD is a risk factor for many metabolic syndromes—including insulin resistance, type 2 diabetes, and cardiovascular disease—the development of which results in increased morbidity and mortality [2,10].

**Figure 1:**
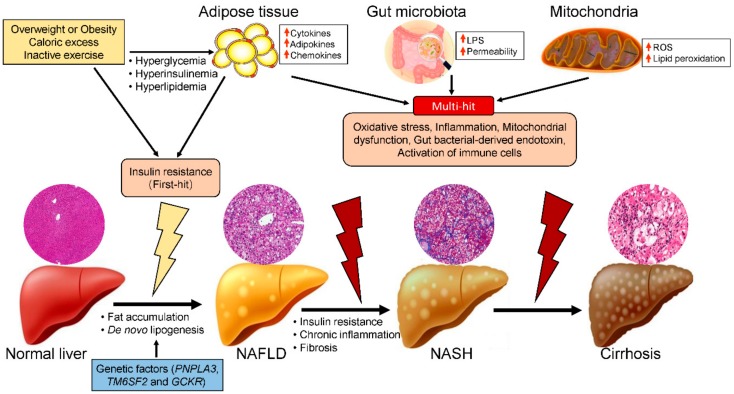
Hypothetical mechanism of NAFLD/NASH progression. Single-nucleotide polymorphisms in several genes—including those encoding patatin-like phospholipase domain-containing 3 (*PNPLA3*), transmembrane 6 superfamily 2 (*TM6SF2*), and glucokinase regulatory protein (*GCKR*)—play important roles in the pathogenesis of NAFLD. Excess caloric consumption and/or physical inactivity induce hyperglycemia, hyperinsulinemia, and high levels of proinflammatory factors, leading to insulin resistance (first hit), and subsequently simple fatty liver (NAFLD). This is followed by other hits, including oxidative stress, chronic inflammation, and the activation of immune cells due to proinflammatory cytokines, the gut microbiota, and mitochondrial dysfunction. These factors cause simple fatty liver to deteriorate to steatohepatitis (NASH) and ultimately hepatic cirrhosis.

NAFLD is caused by factors other than alcohol and other damaging agents, such as hepatitis C, medications, parenteral nutrition, Wilson’s disease, and severe malnutrition [11], and is diagnosed when hepatic fat accumulation in the form of triglycerides (TG) exceeds 5% of the total liver weight [12]. NAFLD is a genetic-environmental-metabolic stress-related disease of unclear pathogenesis. The two-hit hypothesis of NASH progression was proposed by Day et al. [13,14]. The first hit is hepatocellular lipid accumulation resulting from uptake of circulating free fatty acids (FFAs), de novo lipogenesis, and dietary fat. The second hit is steatohepatitis resulting from oxidative stress and proinflammatory factors. Alternatively, the multiple-hit-hypothesis suggests that other factors, including adipokines and mitochondrial dysfunction, also contribute to the development of NAFLD (Figure 1) [15,16]. Thus, here we describe the role of mitochondria in the progression of NASH and the beneficial effect of glucoraphanin on its progression, which is mediated by improvement of mitochondrial dysfunction.

## 2. Risk Factors for NASH

Obesity, insulin resistance, oxidative stress, and inflammatory cascades are thought to play central roles in the pathogenesis of NAFLD (Figure 1) [17]. Obesity leads to insulin resistance and hepatic steatosis, which triggers hepatocellular apoptosis, necrosis, inflammatory cell infiltration, and hepatic fibrosis [18]. In patients with obesity, hypertrophic adipocytes produce several cytokines and chemokines, such as interleukin (IL)-6, tumor necrosis factor-α (TNF-α), IL-1β, and monocyte chemoattractant protein-1 (MCP-1) [19,20,21]. These proinflammatory cytokines and chemokines downregulate hepatic insulin sensitivity by enhancing proinflammatory signaling and attenuating insulin receptor signaling, resulting in hepatic insulin resistance [22]. This insulin resistance is the key factor in the development of NAFLD. According to the two-hit hypothesis, insulin resistance (the first hit) impairs the suppression of lipolysis in adipose tissue and increases the levels of circulating FFAs [23,24]. High levels of FFAs in peripheral blood flux into the liver, resulting in the hypersynthesis of lipids and excessive hepatic lipid accumulation.

Another important factor contributing to insulin resistance is the recruitment of immune cells, including macrophages and T cells, into adipose tissue [22,25]. Notably, adipose tissue macrophages (ATMs) are essential for the pathogenesis of obesity and insulin resistance, both in genetic and diet-induced obese rodents and in patients with obesity [26]. In response to various environmental signals, macrophages may polarize to classical macrophages (M1) following stimulation by lipopolysaccharide (LPS), interferon (IFN)-γ, and TNFα, or to alternative macrophages (M2) following stimulation by IL-4 and transforming growth factor-β1 (TGF β1) [27]. M1-polarized macrophages are key factors in a variety of chronic inflammatory diseases, such as insulin resistance, diabetes, atherosclerosis, and NASH, which are associated with obesity [28,29]. Miura et al. demonstrated that infiltration of Ly6C^+^ bone-marrow-derived macrophages promotes NASH after 22 weeks of a high-fat diet [30]. The effects of proinflammatory cytokines produced by M1 macrophages are counterbalanced by M2-polarized macrophages, which promote resolution of inflammation and tissue repair [26]. M2 macrophages attenuate the development of several inflammatory disorders, including insulin resistance, diabetes, and NASH [31,32].

Hepatic macrophages, which consist of resident Kupffer cells (KCs) and recruited bone-marrow-derived macrophages, are the major cells that produce proinflammatory mediators such as TNFα and IL-1β, which cause systemic insulin resistance as well as NASH [33,34]. Like ATMs, KCs are also phenotypically divided into M1 macrophages, which are induced by Toll-like receptor ligands, such as LPS and IFN-γ, and M2 macrophages, the production of which is triggered by IL-4/IL-13 [28,34]. In the progression of NASH, KCs express membrane receptors and produce excessive levels of cytokines, chemokines, peroxide, and nitric oxide [35]. The proinflammatory cytokines produced by KCs recruit hepatic T lymphocytes and natural killer cells [36]. Dysregulation of M1/M2-KC/macrophage polarization is a key factor in the pathogenesis of chronic inflammation and comorbidities such as insulin resistance and NAFLD [35,36]. Wan et al. demonstrated that M2 KCs/macrophages protect against alcoholic and nonalcoholic fatty liver disease by promoting the apoptosis of M1 macrophages/KCs [31]. This implies that strategies for suppressing M1 macrophage polarization and/or enhancing M2 macrophage polarization may protect against chronic inflammation and insulin resistance, and thereby attenuate the progression of NASH.

Unhealthy-lifestyle-related obesity, insulin resistance, and diabetes are the main causes of NASH. The major lifestyle interventions are diet and exercise, with both dietary restriction and physical activity shown to reduce the risk of developing insulin resistance and NAFLD [37,38]. NASH is also a genetic disease; certain genes—including those encoding patatin-like phospholipase domain-containing 3 (*PNPLA3*), transmembrane 6 superfamily 2 (*TM6SF2*), and glucokinase regulatory protein (*GCKR*)—have been implicated as predisposing to its progression to NASH (Figure 1) [39,40]. Genome-wide association studies have revealed that several single-nucleotide polymorphisms (SNPs) are associated with the pathology of NAFLD. The *PNPLA3* gene variant I148M showed a strong relationship with the development and progression of NAFLD/NASH, and with NAFLD-related cirrhosis [41,42,43]. The *TM6SF2* gene variant E167K is also associated with NAFLD, and is related to cardiovascular disease development [44]. *GCKR* encodes a hepatocyte-specific inhibitor of the glucose-metabolizing enzyme glucokinase in the fasting state [45]. After a meal, hepatic glucokinase is released to the cytoplasm and stimulates glycogen deposition and de novo lipogenesis, resulting in NAFLD and NASH.

There is an updated hypothesis on the role of the microbiota composition in the onset and progression of obesity and NAFLD (Figure 1) [46]. The alteration of the intestinal microbiota composition and barrier function result in increased permeation of bacterial endotoxin, a contributor to NAFLD [47]. Serum levels of endotoxin are higher in patients with NAFLD than in normal individuals. Moreover, attenuating the activation of endotoxin receptor protects against the onset and progression of NAFLD in animal models. Indeed, gut-derived bacteria activate inflammation and promote the synthesis of proinflammatory cytokines in the liver, which play a critical role in the progression of NAFLD [47].

## 3. Roles of Mitochondrial Dysfunction in the Pathogenesis of NASH

**Figure 2:**
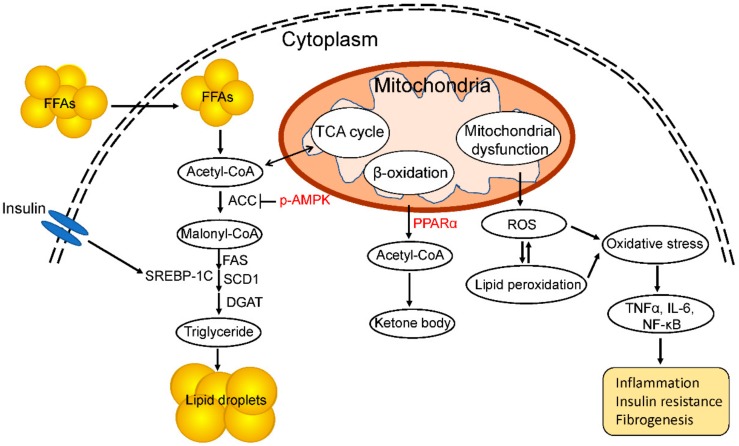
Role of mitochondrial dysfunction in the progression of NAFLD and NASH. De novo lipogenesis plays a critical role in the development of NASH. Acetyl-CoA is a substrate for fatty-acid synthesis, and the expression of SREBP-1c and its target genes encoding lipogenic enzymes is induced by circulating insulin. Phosphorylation of AMP-activated protein kinase (AMPK) inhibits the activity of acetyl CoA carboxylase (ACC), decreasing fat accumulation. Peroxisome proliferator-activated receptor (PPAR) α catalyzes fatty-acid β-oxidation in mitochondria. Mitochondrial dysfunction increases ROS production and lipid peroxidation, leading to a high level of oxidative stress and chronic inflammation and fibrosis of the liver.

Mitochondria are double-membraned organelles that are present in nearly all eukaryotic cells, in which they generate adenosine triphosphate (ATP) using substrates derived from fats and carbohydrates. In addition to energy production, mitochondria have been implicated in various physiologic processes, including the production of reactive oxygen species (ROS), lipid metabolism, regulation of cellular levels of substrates, apoptosis, metal metabolism (e.g., the Fe–S cluster), calcium homeostasis and flux, heat production, and insulin secretion [48]. Due to the importance of mitochondria in cellular energy metabolism, defects in the processes mentioned above have important outcomes at the tissue and systemic levels. Therefore, the dysfunction of mitochondria can have severe consequences (Figure 2) [49].

The multiple-hit hypothesis suggests that mitochondrial dysfunction plays a critical role in the pathogenesis of NAFLD (Figure 1). Impaired mitochondrial function not only impacts hepatic lipid metabolism but also leads to a high level of ROS, triggering lipid peroxidation, cytokine production, and cell death (Figure 2) [50,51]. Hepatocytes are normally rich in mitochondria, which play a central role in their metabolism, being the primary site of fatty-acid oxidation and oxidative phosphorylation. Fatty-acid oxidation for energy production takes place in the liver during long-term fasting and high-intensity physical activity [52]. Fatty-acid β-oxidation in mitochondria is the most efficient means of energy production in metabolic tissues, such as the liver, heart, and muscle, while glucose oxidation, glycolysis, lactate, and ketones also contribute to ATP production [53]. Carnitine palmitoyl transferase I (CPT-I) is the master controller of the hepatic mitochondrial β-oxidation flux. Enhancement of CPT-I activity has been reported to protect against the development of NAFLD [54]. In contrast, downregulation of CPT-I expression in mitochondria impairs fatty-acid oxidation, leading to the development of NASH [55].

De novo lipogenesis is the process by which a cell converts excess carbohydrates into fatty acids via acetyl-coenzyme A (acetyl-CoA) (Figure 2) [56]. The uptake of carbohydrates increases the serum insulin concentration, promoting de novo lipogenesis. Carbohydrate metabolism results in an increased level of acetyl-CoA, which is used as a substrate for lipogenesis. Moreover, hyperinsulinemia leads to activation of the key transcription factor sterol regulatory element-binding protein-1c (SREBP-1c), which regulates hepatic triglyceride synthesis and contributes to steatosis [57]. SREBP-1c is the master regulator of the expression of lipogenic genes and is regulated by insulin through a phosphoinositide 3-kinase (PI3K)-dependent mechanism involving liver X receptor α (LXRα). This induces the expression of SREBP-1c and its target genes, including those encoding fatty-acid synthase (FAS), acetyl CoA carboxylase (ACC), and stearoyl-CoA desaturase (SCD1) (Figure 2) [58,59]. Carbohydrate-responsive element-binding protein (ChREBP) is another major transcriptional regulator that induces the synthesis of key enzymes responsible for hepatic lipogenesis [60]. Insulin resistance or hyperinsulinemia drives de novo lipogenesis by increasing the production and activation of SREBP-1c and carbohydrate regulatory element binding protein (ChREBP), and enhancing acetyl CoA carboxylase (ACC) activity, further increasing hepatic lipid accumulation [56]. Glycolysis generates pyruvate, which is transformed in mitochondria into acetyl-CoA and citrate. In the cytosol, acetyl-CoA is transformed to malonyl-CoA and then to palmitate by ACC and FAS, respectively. Moreover, a high malonyl-CoA level inhibits CPT-I and decreases fatty-acid β-oxidation [61]. Therefore, fatty acids are not degraded, but are instead directed towards the formation of triglycerides, which are secreted as very low-density lipoprotein (VLDL). Moreover, incorrect protein folding; e.g., of apoB, which is essential for VLDL, may impair lipid export from the liver and exacerbate steatosis in mice [62]. In addition, AMP-activated protein kinase (AMPK) phosphorylates ACC1 and ACC2, reducing ACC activity and decreasing malonyl-CoA levels, leading to inhibition of de novo lipogenesis and increasing mitochondrial fatty-acid oxidation (Figure 2) [63,64]. Furthermore, AMPK downregulates the expression of lipogenesis-related genes by directly phosphorylating the master transcriptional regulator of lipogenesis SREBP-1c [65]. Thus, the activation of AMPK protects against NAFLD by suppressing de novo lipogenesis, increasing fatty-acid oxidation, and enhancing mitochondrial function in the liver.

Peroxisome proliferator-activated receptor (PPAR) α is also essential for glucagon-mediated fatty-acid oxidation [66]. The PPAR subfamily contains two other isotypes, PPARβ/δ and PPARγ, each of which has a specific tissue distribution and function. PPARα expression is enriched in tissues with active fatty-acid oxidation, such as the liver, heart, and skeletal muscle, and it serves as a nutritional sensor, enabling adaptation of the rates of fat catabolism, lipogenesis, and ketone body synthesis in response to feeding and fasting [67]. PPARα regulates the transcription of genes related to peroxisomal and mitochondrial β-oxidation, fatty-acid transport, and hepatic glucose production, the latter being rodent-specific (Figure 2). PPARα negatively regulates chronic inflammation-related metabolic syndromes, including obesity, atherosclerosis, and NASH [68]. Moreover, a selective PPARα agonist increased fatty-acid oxidation, improved insulin resistance and the energy balance, and decreased body weight in an animal model [69]. Furthermore, PPARα modulated the transcription of FGF21, thereby attenuating hepatic insulin resistance and hepatic steatosis in a mouse model [70].

Mitochondria undertake electron transport to generate ATP, and are an important source of the ROS that contributes to NASH [71]. Dysfunction of the mitochondrial respiratory chain can lead to excess production of ROS. Mitochondrial abnormalities alter the balance between pro- and anti-oxidant mechanisms, leading to an increase in the levels of nonmetabolized fatty acids in the cytosol as a result of the blockade of fatty-acid β-oxidation and the consequent induction of ROS production [72]. The higher production of ROS under lipid-rich conditions causes lipid peroxidation and generates reactive aldehydic derivatives (e.g., Malondialdehyde, MDA), which exert detrimental effects on hepatocytes and other liver cells. The products of lipid peroxidation and ROS directly attack and inactivate the respiratory chain in hepatocytes, which further increases the generation of ROS [73]. Indeed, excess production of ROS in mitochondria has been demonstrated in mice or rats fed a choline-deficient diet, an animal model of steatohepatitis [74]. Moreover, animal models of NASH feature extensive lipid peroxidation [75]. In addition, both ROS and lipid peroxidation products attack mitochondrial DNA (mtDNA), reducing mitochondrial number and function and leading to the development of steatosis and liver lesions [76]. ROS in mitochondria activate the nuclear factor-kappaB pathway, inducing TNF-α expression and consequently mtDNA damage. An increased level of 8-hydroxy-2′deoxyguanosine, an indicator of ROS-mediated mtDNA damage, is correlated with the grade of inflammation and with NAFLD/NASH [76]. Notably, mitochondrial dysfunction not only facilitates the production of ROS, but also contributes to the progression of NAFLD/NASH by inducing the hepatic production of proinflammatory cytokines, including TNFα, IL-1β, and IL-6, which are the major contributors to the second hit [77].

## 4. The Nrf2-Keap1 Pathway in NASH

As stated above, the generation of oxidative stress by defective mitochondria is closely associated with the development of NAFLD/NASH. Nuclear factor (erythroid derived 2)-like 2 (Nrf2), a basic leucine zipper transcription factor, is expressed in most human and mouse tissues as a defense response to extrinsic and intrinsic stressors [78]. When ROS generation and/or lipid peroxidation occur in mitochondria, kelch-like ECH-associated protein 1 (Keap1) senses cellular oxidative stress and releases Nrf2, leading to increased levels of free Nrf2 and Nrf2 nuclear translocation [79]. Nuclear Nrf2 binds to the consensus nucleotide sequence, an antioxidant response element, in the promoter regions of a battery of genes that encode antioxidant enzymes [79]. The target antioxidants include nicotinamide adenine dinucleotide phosphate (NADPH)-oxidase, quinone oxidoreductase 1, hemeoxygenase-1, glutathione S-transferase, superoxide dismutase, catalase, and γ-glutamate cysteine ligase [79]. In this manner, Nrf2 defends against the cytotoxic effects of oxidative stress. Moreover, inducers of Nrf2—such as the triterpenoid 2-cyano-3,12-dioxoolean-1,9-dien-28-oic acid (CDDO)-imidazolide, CDDO-methyl ester (also known as bardoxolone methyl), and the dithiolethione analog, oltipraz—reportedly attenuate oxidative stress and ameliorate obesity-related diseases, including diabetes and hepatic steatosis [80,81,82]. 

## 5. Impact of Glucoraphanin on Mitochondrial Dysfunction-Related NASH

Sulforaphane, an isothiocyanate derived from cruciferous vegetables, is one of the most potent naturally occurring inducers of Nrf2. This compound mediates a xenobiotic response to predation via the vesicular release of the hydrolytic enzyme myrosinase from damaged cells; this enzyme converts glucosinolates to isothiocyantes [83,84]. Over the last two decades, sulforaphane has been extensively characterized in terms of its anticancer, antioxidant, and antimicrobial properties. Much of the activity of sulforaphane has been attributed to its ability to modulate the KEAP1-Nrf2-antioxidant response element signaling pathway [85]. Among cruciferous vegetables, broccoli sprouts are the best source of glucoraphanin, a stable glucosinolate precursor of sulforaphane [86]. In both rodents and humans, glucoraphanin is hydrolyzed by gut microbiota-derived myrosinase into bioactive sulforaphane, which is absorbed from the intestine [86]. Induction of the expression of Nrf2, as well as global Nrf2 knockout and Keap1 knockdown, protects against high-fat diet (HFD)-induced obesity and related comorbidities [87,88]. Nagata et al. showed that glucoraphanin decreased body-weight gain and attenuated hepatic steatosis in HFD-fed mice [89]. In obese mice, glucoraphanin increased energy expenditure and the level of uncoupling protein 1, implying improved mitochondrial function. Additionally, glucoraphanin reduced hepatic lipogenesis and lipid peroxidation, but elevated hepatic β-oxidation, resulting in attenuation of obesity-related NAFLD [89]. Therefore, by increasing energy expenditure, limiting chronic inflammation, and modulating redox stress, glucoraphanin may mitigate obesity, insulin resistance, and NAFLD.

Nrf2 is the master regulator of cellular redox homeostasis and counterbalances mitochondrial ROS production [78]. In addition to overall cellular redox homeostasis, Nrf2 is also critical for maintaining mitochondrial redox homeostasis [79,90,91]. Increasing the expression of Nox2, the catalytic subunit of NADPH oxidase (NOX) increased the rate of ROS production in Nrf2-deficient cells and tissues [92]. Indeed, the absence of Nrf2 impaired mitochondrial complex I activity, leading to increased ROS production [92]. Moreover, the NOX-dependent activation of Nrf2 is an important endogenous mechanism for protection against mitochondrial damage and cell death in the heart during chronic pressure overload [93]. Furthermore, Nrf2 function is reported to be impaired in mitochondria-related disorders, whereas Nrf2 activation has beneficial effects [90]. Carrasco-Pozo et al. reported that sulforaphane improved mitochondrial bioenergetic function and protected against cholesterol-induced pancreatic β-cell dysfunction, enabling control of hyperglycemia, in a mouse model [94]. Moreover, sulforaphane preserves mitochondrial function and limits lipid peroxidation and seizure-induced neuronal degeneration in a mouse model of acute seizure [95]. Furthermore, a diet rich in high-glucoraphanin broccoli modulated mitochondrial function and ameliorated the perturbation of the plasma metabolite profile [96]. Therefore, the induction of Nrf2 by sulforaphane, or its precursor glucoraphanin, preserves mitochondrial function, implying that it may protect against the development of NASH.

## 6. Concluding Remarks and Future Directions

The prevalence of NAFLD is increasing worldwide due to the increase in the number of obese individuals. NAFLD is caused by the impaired regulation of hepatic lipid and glucose homeostasis. Unfortunately, the sequence of events that occurs during the pathogenesis of NAFLD is unclear. A variety of factors—including insulin resistance, cytokines, oxidative stress, the microbiota, and mitochondrial dysfunction—are involved in the pathogenesis of NAFLD. To adapt to lipid accumulation, hepatic mitochondria increase fatty-acid β-oxidation and electron-transport-chain activity, resulting in ROS generation. Oxidative stress due to ROS plays a key role in the development of insulin resistance and NASH. Therefore, therapeutic approaches have focused on antioxidant compounds to counteract ROS. Studies involving NAFLD mice have shown that lipotoxicity-induced insulin resistance and hepatic steatosis can be prevented by antioxidants. Moreover, these compounds can ameliorate pathological features such as inflammation and hepatic fibrosis, which are typical of advanced NAFLD. Notably, the anti-obesity agents sulforaphane and glucoraphanin prevent hepatic steatosis by increasing energy utilization and preventing lipogenesis and oxidative stress in the liver. Moreover, they improve mitochondrial dysfunction. Thus, future studies are needed to determine the effect of sulforaphane and glucoraphanin on mitochondrial dysfunction in NAFLD.

## Abbreviations

NAFLD	Non-alcoholic fatty liver disease
NASH	Non-alcoholic steatohepatitis
ROS	Reactive oxygen species
Nrf	Nuclear factor erythroid 2-related factor
TG	Triglycerides
IL	Interleukin
MCP	Monocyte chemoattractant protein
TNF	Tumor necrosis factor
FFA	Free fatty acid
LPS	Lipopolysaccharide
IFN	Interferon
ATM	Adipose tissue macrophage
KC	Kupffer cell
PNPLA3	Patatin-like phospholipase domain-containing 3
TM6SF2	Transmembrane 6 superfamily 2
GCKR	Glucokinase regulatory protein
SNP	Single-nucleotide polymorphism
ATP	Adenosine triphosphate
CPT	Carnitine palmitoyl transferase
SREBP	Sterol regulatory element-binding protein
PI3K	Phosphoinositide 3-kinase
LXR	Liver X receptor
FAS	Fatty-acid synthase
ACC	Acetyl CoA carboxylase
SCD	Stearoyl-CoA desaturase
ChREBP	Carbohydrate-responsive element-binding protein
VLDL	Very low-density lipoprotein
AMPK	AMP-activated protein kinase
PPAR	Peroxisome proliferator-activated receptor
mtDNA	Mitochondrial DNA
Keap1	kelch-like ECH-associated protein 1
HFD	High-fat diet
NOX	NADPH oxidase
